# Supplementary material for: Resolving the full spectrum of human genome variation using Linked-Reads
Source: Genome Res. 2019 Apr;29(4):635–45. doi: 10.1101/gr.234443.118 (PMC6442396; doi:10.1101/gr.234443.118)
Supplement: Supplemental Material [file supp_gr.234443.118_Supplemental_longranger-2.2.zip › longranger-2.2.tar.bz2!/lr2.2-public/lib/stan/cmdstan/stan/doc/api/html/weibull_8hpp_source.html]

Stan: src/stan/prob/distributions/univariate/continuous/weibull.hpp Source File


|  |  |
| --- | --- |
|  | Stan  2.5.0  probability, sampling & optimization |


- Main Page
- Related Pages
- Namespaces
- Classes
- Files

- File List
- File Members

All Classes Namespaces Files Functions Variables Typedefs Enumerations Enumerator Friends Macros Pages

- src
- stan
- prob
- distributions
- univariate
- continuous

weibull.hpp

Go to the documentation of this file.

1 #ifndef STAN\_\_PROB\_\_DISTRIBUTIONS\_\_WEIBULL\_HPP

2 #define STAN\_\_PROB\_\_DISTRIBUTIONS\_\_WEIBULL\_HPP

3

4 #include <boost/random/weibull\_distribution.hpp>

5 #include <boost/random/variate\_generator.hpp>

6

7 #include <stan/agrad/partials\_vari.hpp>

8 #include <stan/math/error\_handling.hpp>

9 #include <stan/math/functions/multiply\_log.hpp>

10 #include <stan/math/functions/value\_of.hpp>

11 #include <stan/meta/traits.hpp>

12 #include <stan/prob/constants.hpp>

13 #include <stan/prob/traits.hpp>

14

15 namespace stan {

16

17  namespace prob {

18

19  // Weibull(y|alpha,sigma) [y >= 0; alpha > 0; sigma > 0]

20  // FIXME: document

21  template <bool propto,

22  typename T\_y, typename T\_shape, typename T\_scale>

23  typename return\_type<T\_y,T\_shape,T\_scale>::type

24  weibull\_log(const T\_y& y, const T\_shape& alpha, const T\_scale& sigma) {

25  static const char\* function = "stan::prob::weibull\_log(%1%)";

26

27  using stan::math::check\_positive\_finite;

28  using stan::math::check\_finite;

29  using stan::math::check\_not\_nan;

30  using stan::math::value\_of;

31  using stan::math::check\_consistent\_sizes;

32  using stan::math::multiply\_log;

33

34  // check if any vectors are zero length

35  if (!(stan::length(y)

36  && stan::length(alpha)

37  && stan::length(sigma)))

38  return 0.0;

39

40  // set up return value accumulator

41  double logp(0.0);

42  check\_finite(function, y, "Random variable", &logp);

43  check\_positive\_finite(function, alpha, "Shape parameter", &logp);

44  check\_positive\_finite(function, sigma, "Scale parameter", &logp);

45  check\_consistent\_sizes(function,

46  y,alpha,sigma,

47  "Random variable","Shape parameter",

48  "Scale parameter",

49  &logp);

50

51  // check if no variables are involved and prop-to

52  if (!include\_summand<propto,T\_y,T\_shape,T\_scale>::value)

53  return 0.0;

54

55  VectorView<const T\_y> y\_vec(y);

56  VectorView<const T\_shape> alpha\_vec(alpha);

57  VectorView<const T\_scale> sigma\_vec(sigma);

58  size\_t N = max\_size(y, alpha, sigma);

59

60  for (size\_t n = 0; n < N; n++) {

61  const double y\_dbl = value\_of(y\_vec[n]);

62  if (y\_dbl < 0)

63  return LOG\_ZERO;

64  }

65

66  DoubleVectorView<include\_summand<propto,T\_shape>::value,

67  is\_vector<T\_shape>::value> log\_alpha(length(alpha));

68  for (size\_t i = 0; i < length(alpha); i++)

69  if (include\_summand<propto,T\_shape>::value)

70  log\_alpha[i] = log(value\_of(alpha\_vec[i]));

71

72  DoubleVectorView<include\_summand<propto,T\_y,T\_shape>::value,

73  is\_vector<T\_y>::value> log\_y(length(y));

74  for (size\_t i = 0; i < length(y); i++)

75  if (include\_summand<propto,T\_y,T\_shape>::value)

76  log\_y[i] = log(value\_of(y\_vec[i]));

77

78  DoubleVectorView<include\_summand<propto,T\_shape,T\_scale>::value,

79  is\_vector<T\_scale>::value> log\_sigma(length(sigma));

80  for (size\_t i = 0; i < length(sigma); i++)

81  if (include\_summand<propto,T\_shape,T\_scale>::value)

82  log\_sigma[i] = log(value\_of(sigma\_vec[i]));

83

84  DoubleVectorView<include\_summand<propto,T\_y,T\_shape,T\_scale>::value,

85  is\_vector<T\_scale>::value> inv\_sigma(length(sigma));

86  for (size\_t i = 0; i < length(sigma); i++)

87  if (include\_summand<propto,T\_y,T\_shape,T\_scale>::value)

88  inv\_sigma[i] = 1.0 / value\_of(sigma\_vec[i]);

89

90  DoubleVectorView<include\_summand<propto,T\_y,T\_shape,T\_scale>::value,

91  is\_vector<T\_y>::value | is\_vector<T\_shape>::value | is\_vector<T\_scale>::value>

92  y\_div\_sigma\_pow\_alpha(N);

93  for (size\_t i = 0; i < N; i++)

94  if (include\_summand<propto,T\_y,T\_shape,T\_scale>::value) {

95  const double y\_dbl = value\_of(y\_vec[i]);

96  const double alpha\_dbl = value\_of(alpha\_vec[i]);

97  y\_div\_sigma\_pow\_alpha[i] = pow(y\_dbl \* inv\_sigma[i], alpha\_dbl);

98  }

99

100  agrad::OperandsAndPartials<T\_y,T\_shape,T\_scale> operands\_and\_partials(y,alpha,sigma);

101  for (size\_t n = 0; n < N; n++) {

102  const double alpha\_dbl = value\_of(alpha\_vec[n]);

103  if (include\_summand<propto,T\_shape>::value)

104  logp += log\_alpha[n];

105  if (include\_summand<propto,T\_y,T\_shape>::value)

106  logp += (alpha\_dbl-1.0)\*log\_y[n];

107  if (include\_summand<propto,T\_shape,T\_scale>::value)

108  logp -= alpha\_dbl\*log\_sigma[n];

109  if (include\_summand<propto,T\_y,T\_shape,T\_scale>::value)

110  logp -= y\_div\_sigma\_pow\_alpha[n];

111

112  if (!is\_constant\_struct<T\_y>::value) {

113  const double inv\_y = 1.0 / value\_of(y\_vec[n]);

114  operands\_and\_partials.d\_x1[n]

115  += (alpha\_dbl-1.0) \* inv\_y

116  - alpha\_dbl \* y\_div\_sigma\_pow\_alpha[n] \* inv\_y;

117  }

118  if (!is\_constant\_struct<T\_shape>::value)

119  operands\_and\_partials.d\_x2[n]

120  += 1.0/alpha\_dbl

121  + (1.0 - y\_div\_sigma\_pow\_alpha[n]) \* (log\_y[n] - log\_sigma[n]);

122  if (!is\_constant\_struct<T\_scale>::value)

123  operands\_and\_partials.d\_x3[n]

124  += alpha\_dbl \* inv\_sigma[n] \* ( y\_div\_sigma\_pow\_alpha[n] - 1.0 );

125  }

126  return operands\_and\_partials.to\_var(logp);

127  }

128

129  template <typename T\_y, typename T\_shape, typename T\_scale>

130  inline

131  typename return\_type<T\_y,T\_shape,T\_scale>::type

132  weibull\_log(const T\_y& y, const T\_shape& alpha, const T\_scale& sigma) {

133  return weibull\_log<false>(y,alpha,sigma);

134  }

135

136  template <typename T\_y, typename T\_shape, typename T\_scale>

137  typename return\_type<T\_y,T\_shape,T\_scale>::type

138  weibull\_cdf(const T\_y& y, const T\_shape& alpha, const T\_scale& sigma) {

139

140  static const char\* function = "stan::prob::weibull\_cdf(%1%)";

141

142  using stan::math::check\_positive\_finite;

143  using stan::math::check\_nonnegative;

144  using boost::math::tools::promote\_args;

145  using stan::math::value\_of;

146

147  // check if any vectors are zero length

148  if (!(stan::length(y)

149  && stan::length(alpha)

150  && stan::length(sigma)))

151  return 1.0;

152

153  double cdf(1.0);

154  check\_nonnegative(function, y, "Random variable", &cdf);

155  check\_positive\_finite(function, alpha, "Shape parameter", &cdf);

156  check\_positive\_finite(function, sigma, "Scale parameter", &cdf);

157

158  agrad::OperandsAndPartials<T\_y, T\_shape, T\_scale>

159  operands\_and\_partials(y, alpha, sigma);

160

161  VectorView<const T\_y> y\_vec(y);

162  VectorView<const T\_scale> sigma\_vec(sigma);

163  VectorView<const T\_shape> alpha\_vec(alpha);

164  size\_t N = max\_size(y, sigma, alpha);

165  for (size\_t n = 0; n < N; n++) {

166  const double y\_dbl = value\_of(y\_vec[n]);

167  const double sigma\_dbl = value\_of(sigma\_vec[n]);

168  const double alpha\_dbl = value\_of(alpha\_vec[n]);

169  const double pow\_ = pow(y\_dbl / sigma\_dbl, alpha\_dbl);

170  const double exp\_ = exp(-pow\_);

171  const double cdf\_ = 1.0 - exp\_;

172

173  //cdf

174  cdf \*= cdf\_;

175

176  //gradients

177  const double rep\_deriv = exp\_ \* pow\_ / cdf\_;

178  if (!is\_constant\_struct<T\_y>::value)

179  operands\_and\_partials.d\_x1[n] += rep\_deriv \* alpha\_dbl / y\_dbl;

180  if (!is\_constant\_struct<T\_shape>::value)

181  operands\_and\_partials.d\_x2[n] += rep\_deriv \* log(y\_dbl / sigma\_dbl);

182  if (!is\_constant\_struct<T\_scale>::value)

183  operands\_and\_partials.d\_x3[n] -= rep\_deriv \* alpha\_dbl / sigma\_dbl;

184  }

185

186  if (!is\_constant\_struct<T\_y>::value)

187  for (size\_t n = 0; n < stan::length(y); ++n)

188  operands\_and\_partials.d\_x1[n] \*= cdf;

189  if (!is\_constant\_struct<T\_shape>::value)

190  for (size\_t n = 0; n < stan::length(alpha); ++n)

191  operands\_and\_partials.d\_x2[n] \*= cdf;

192  if (!is\_constant\_struct<T\_scale>::value)

193  for (size\_t n = 0; n < stan::length(sigma); ++n)

194  operands\_and\_partials.d\_x3[n] \*= cdf;

195

196  return operands\_and\_partials.to\_var(cdf);

197  }

198

199  template <typename T\_y, typename T\_shape, typename T\_scale>

200  typename return\_type<T\_y,T\_shape,T\_scale>::type

201  weibull\_cdf\_log(const T\_y& y, const T\_shape& alpha, const T\_scale& sigma) {

202

203  static const char\* function = "stan::prob::weibull\_cdf\_log(%1%)";

204

205  using stan::math::check\_positive\_finite;

206  using stan::math::check\_nonnegative;

207  using boost::math::tools::promote\_args;

208  using stan::math::value\_of;

209

210  // check if any vectors are zero length

211  if (!(stan::length(y)

212  && stan::length(alpha)

213  && stan::length(sigma)))

214  return 0.0;

215

216  double cdf\_log(0.0);

217  check\_nonnegative(function, y, "Random variable", &cdf\_log);

218  check\_positive\_finite(function, alpha, "Shape parameter", &cdf\_log);

219  check\_positive\_finite(function, sigma, "Scale parameter", &cdf\_log);

220

221  agrad::OperandsAndPartials<T\_y, T\_shape, T\_scale>

222  operands\_and\_partials(y, alpha, sigma);

223

224  VectorView<const T\_y> y\_vec(y);

225  VectorView<const T\_scale> sigma\_vec(sigma);

226  VectorView<const T\_shape> alpha\_vec(alpha);

227  size\_t N = max\_size(y, sigma, alpha);

228  for (size\_t n = 0; n < N; n++) {

229  const double y\_dbl = value\_of(y\_vec[n]);

230  const double sigma\_dbl = value\_of(sigma\_vec[n]);

231  const double alpha\_dbl = value\_of(alpha\_vec[n]);

232  const double pow\_ = pow(y\_dbl / sigma\_dbl, alpha\_dbl);

233  const double exp\_ = exp(-pow\_);

234  const double cdf\_ = 1.0 - exp\_;

235

236  //cdf\_log

237  cdf\_log += log(cdf\_);

238

239  //gradients

240  const double rep\_deriv = pow\_ / (1.0 / exp\_ - 1.0);

241  if (!is\_constant\_struct<T\_y>::value)

242  operands\_and\_partials.d\_x1[n] += rep\_deriv \* alpha\_dbl / y\_dbl;

243  if (!is\_constant\_struct<T\_shape>::value)

244  operands\_and\_partials.d\_x2[n] += rep\_deriv \* log(y\_dbl / sigma\_dbl);

245  if (!is\_constant\_struct<T\_scale>::value)

246  operands\_and\_partials.d\_x3[n] -= rep\_deriv \* alpha\_dbl / sigma\_dbl;

247  }

248

249  return operands\_and\_partials.to\_var(cdf\_log);

250  }

251

252  template <typename T\_y, typename T\_shape, typename T\_scale>

253  typename return\_type<T\_y,T\_shape,T\_scale>::type

254  weibull\_ccdf\_log(const T\_y& y, const T\_shape& alpha, const T\_scale& sigma) {

255

256  static const char\* function = "stan::prob::weibull\_ccdf\_log(%1%)";

257

258  using stan::math::check\_positive\_finite;

259  using stan::math::check\_nonnegative;

260  using boost::math::tools::promote\_args;

261  using stan::math::value\_of;

262

263  // check if any vectors are zero length

264  if (!(stan::length(y)

265  && stan::length(alpha)

266  && stan::length(sigma)))

267  return 0.0;

268

269  double ccdf\_log(0.0);

270  check\_nonnegative(function, y, "Random variable", &ccdf\_log);

271  check\_positive\_finite(function, alpha, "Shape parameter", &ccdf\_log);

272  check\_positive\_finite(function, sigma, "Scale parameter", &ccdf\_log);

273

274  agrad::OperandsAndPartials<T\_y, T\_shape, T\_scale>

275  operands\_and\_partials(y, alpha, sigma);

276

277  VectorView<const T\_y> y\_vec(y);

278  VectorView<const T\_scale> sigma\_vec(sigma);

279  VectorView<const T\_shape> alpha\_vec(alpha);

280  size\_t N = max\_size(y, sigma, alpha);

281  for (size\_t n = 0; n < N; n++) {

282  const double y\_dbl = value\_of(y\_vec[n]);

283  const double sigma\_dbl = value\_of(sigma\_vec[n]);

284  const double alpha\_dbl = value\_of(alpha\_vec[n]);

285  const double pow\_ = pow(y\_dbl / sigma\_dbl, alpha\_dbl);

286

287  //ccdf\_log

288  ccdf\_log -= pow\_;

289

290  //gradients

291  if (!is\_constant\_struct<T\_y>::value)

292  operands\_and\_partials.d\_x1[n] -= alpha\_dbl / y\_dbl \* pow\_;

293  if (!is\_constant\_struct<T\_shape>::value)

294  operands\_and\_partials.d\_x2[n] -= log(y\_dbl / sigma\_dbl) \* pow\_;

295  if (!is\_constant\_struct<T\_scale>::value)

296  operands\_and\_partials.d\_x3[n] += alpha\_dbl / sigma\_dbl \* pow\_;

297  }

298

299  return operands\_and\_partials.to\_var(ccdf\_log);

300  }

301

302  template <class RNG>

303  inline double

304  weibull\_rng(const double alpha,

305  const double sigma,

306  RNG& rng) {

307  using boost::variate\_generator;

308  using boost::random::weibull\_distribution;

309

310  static const char\* function = "stan::prob::weibull\_rng(%1%)";

311

312  using stan::math::check\_positive\_finite;

313

314  check\_positive\_finite(function, alpha, "Shape parameter", (double\*)0);

315  check\_positive\_finite(function, sigma, "Scale parameter", (double\*)0);

316

317  variate\_generator<RNG&, weibull\_distribution<> >

318  weibull\_rng(rng, weibull\_distribution<>(alpha, sigma));

319  return weibull\_rng();

320  }

321  }

322 }

323 #endif

multiply\_log.hpp

traits.hpp

stan::prob::weibull\_rng

double weibull\_rng(const double alpha, const double sigma, RNG &rng)

**Definition:** weibull.hpp:304

stan::prob::weibull\_cdf\_log

return\_type< T\_y, T\_shape, T\_scale >::type weibull\_cdf\_log(const T\_y &y, const T\_shape &alpha, const T\_scale &sigma)

**Definition:** weibull.hpp:201

stan::agrad::OperandsAndPartials::to\_var

T\_return\_type to\_var(double logp)

**Definition:** partials\_vari.hpp:138

stan::math::check\_positive\_finite

bool check\_positive\_finite(const char \*function, const T\_y &y, const char \*name, T\_result \*result)

**Definition:** check\_positive\_finite.hpp:12

stan::agrad::pow

fvar< T > pow(const fvar< T > &x1, const fvar< T > &x2)

**Definition:** pow.hpp:17

error\_handling.hpp

stan::math::multiply\_log

boost::math::tools::promote\_args< T\_a, T\_b >::type multiply\_log(const T\_a a, const T\_b b)

Calculated the value of the first argument times log of the second argument while behaving properly w...

**Definition:** multiply\_log.hpp:51

stan::length

size\_t length(const T &)

**Definition:** traits.hpp:159

stan::prob::weibull\_cdf

return\_type< T\_y, T\_shape, T\_scale >::type weibull\_cdf(const T\_y &y, const T\_shape &alpha, const T\_scale &sigma)

**Definition:** weibull.hpp:138

stan::DoubleVectorView

DoubleVectorView allocates double values to be used as intermediate values.

**Definition:** traits.hpp:358

stan::math::check\_finite

bool check\_finite(const char \*function, const T\_y &y, const char \*name, T\_result \*result)

Checks if the variable y is finite.

**Definition:** check\_finite.hpp:52

partials\_vari.hpp

stan::agrad::value\_of

T value\_of(const fvar< T > &v)

Return the value of the specified variable.

**Definition:** value\_of.hpp:16

constants.hpp

stan::is\_vector

**Definition:** traits.hpp:83

stan::agrad::OperandsAndPartials

A variable implementation that stores operands and derivatives with respect to the variable...

**Definition:** partials\_vari.hpp:76

stan::prob::weibull\_log

return\_type< T\_y, T\_shape, T\_scale >::type weibull\_log(const T\_y &y, const T\_shape &alpha, const T\_scale &sigma)

**Definition:** weibull.hpp:24

traits.hpp

stan::return\_type::type

boost::math::tools::promote\_args< typename scalar\_type< T1 >::type, typename scalar\_type< T2 >::type, typename scalar\_type< T3 >::type, typename scalar\_type< T4 >::type, typename scalar\_type< T5 >::type, typename scalar\_type< T6 >::type >::type type

**Definition:** traits.hpp:406

stan::is\_constant\_struct

Metaprogram to determine if a type has a base scalar type that can be assigned to type double...

**Definition:** traits.hpp:57

stan::math::value\_of

double value\_of(const T x)

Return the value of the specified scalar argument converted to a double value.

**Definition:** value\_of.hpp:24

stan::prob::include\_summand

Template metaprogram to calculate whether a summand needs to be included in a proportional (log) prob...

**Definition:** traits.hpp:35

stan::agrad::OperandsAndPartials::d\_x2

VectorView< double \*, is\_vector< T2 >::value, is\_constant\_struct< T2 >::value > d\_x2

**Definition:** partials\_vari.hpp:83

stan::prob::weibull\_ccdf\_log

return\_type< T\_y, T\_shape, T\_scale >::type weibull\_ccdf\_log(const T\_y &y, const T\_shape &alpha, const T\_scale &sigma)

**Definition:** weibull.hpp:254

stan::math::check\_nonnegative

bool check\_nonnegative(const char \*function, const T\_y &y, const char \*name, T\_result \*result)

**Definition:** check\_nonnegative.hpp:52

stan::math::check\_consistent\_sizes

bool check\_consistent\_sizes(const char \*function, const T1 &x1, const T2 &x2, const char \*name1, const char \*name2, T\_result \*result)

**Definition:** check\_consistent\_sizes.hpp:13

stan::max\_size

size\_t max\_size(const T1 &x1, const T2 &x2)

**Definition:** traits.hpp:191

stan::math::check\_not\_nan

bool check\_not\_nan(const char \*function, const T\_y &y, const char \*name, T\_result \*result)

Checks if the variable y is nan.

**Definition:** check\_not\_nan.hpp:56

value\_of.hpp

stan::agrad::OperandsAndPartials::d\_x1

VectorView< double \*, is\_vector< T1 >::value, is\_constant\_struct< T1 >::value > d\_x1

**Definition:** partials\_vari.hpp:82

stan::agrad::OperandsAndPartials::d\_x3

VectorView< double \*, is\_vector< T3 >::value, is\_constant\_struct< T3 >::value > d\_x3

**Definition:** partials\_vari.hpp:84

stan::agrad::log

fvar< T > log(const fvar< T > &x)

**Definition:** log.hpp:15

stan::VectorView

VectorView is a template metaprogram that takes its argument and allows it to be used like a vector...

**Definition:** traits.hpp:275

stan::agrad::exp

fvar< T > exp(const fvar< T > &x)

**Definition:** exp.hpp:16


---

[ Stan Home Page ]

*© 2011–2014,
Stan Development Team.*
